# Efficacy of Photobiomodulation in the Management of Pain and Inflammation after Dental Implants: A Randomized Clinical Trial

**DOI:** 10.3390/jcm13195709

**Published:** 2024-09-25

**Authors:** Yolanda Collado-Murcia, Francisco Parra-Perez, Pia López-Jornet

**Affiliations:** Department of Dermatology, Stomatology, Radiology and Physical Medicine, Faculty of Medicine, University of Murcia, Hospital Morales Meseguer Clinica Odontologica Marques Velez S/N, 30008 Murcia, Spain; yolanda.cmurcia@gmail.com (Y.C.-M.); pacoppmurcia@gmail.com (F.P.-P.)

**Keywords:** dental implant, pain, inflammation, photobiomodulation

## Abstract

**Background**: Photobiomodulation (PBM) is a non-invasive procedure used to manage pain and inflammation. The aim of this study is to quantitatively measure pain and inflammation and to compare the proposed PBM treatment with a simulated treatment (PBM-SHAM) in patients with dental implants. **Materials and Methods**: A total of 62 patients were included and randomized into two groups. Group 1 (PBM) consisted of 31 patients subjected to the insertion of dental implants and a single intraoral PBM session with an EPIC X Biolase (0.5 W and 15 J/cm²) diode laser. Group II (PBM-SHAM) included 31 patients subjected to dental implants and a simulated PBM. Each patient was given a document with visual analog scales (VASs) to record pain and inflammation during the 7 days post-surgery. The patients were assessed at the end of the week to remove the sutures, to collect the VASs, and to re-evaluate the surveys. **Results**: Through the use of mixed effects models, it was found that the length of time after the surgery and the number of implants placed during the intervention were important variables that had an influence on pain and inflammation. **Conclusions**: PBM is a non-invasive and safe treatment. Postoperative pain and inflammation associated with implant surgery decreased in a similar manner over time, independently of the application of PBM. Therefore, more randomized studies are needed with a standardized methodology to adequately assess the efficacy of this therapy.

## 1. Introduction

Pain, inflammation, and delayed or poor healing are very frequent postoperative complications after surgical procedures in the oral cavity. Therefore, a reduction in these complications is fundamental for the success of any oral surgery [1,2,3,4,5]. The modulation of pain is very complex, as the pain signaling pathways are influenced by physiological and psychological factors of the patient and can vary according the complexity of the procedure, age, gender, the skill of the professional, among other factors [3,4,5,6]. To mitigate these effects and improve the postoperative quality of life, non-steroidal anti-inflammatory drugs (NSAIDs), opioid analgesics, and hygiene and dietary measures are prescribed. Due to the adverse effects of these medications, the use of drugs is limited [3].

Khouly et al. (2021) performed a systematic review to identify randomized controlled clinical trials on postoperative pain and the consumption of rescue analgesics after the placement of a dental implant. They found that pain is usually more severe during the first 72 h after the implant surgery and can be efficiently treated with the short-term use of analgesics [7]. However, given the heterogeneity of the available studies, there was no sufficient evidence to recommend a specific analgesics regimen. The effective management of pain improves the general experience of the patient and determines the success of the procedure. Non-managed pain can increase stress and anxiety, which can negatively impact the healing process and the patient’s compliance with the instructions regarding postoperative self-care.

Recently, new methods have been proposed to reduce pain after surgery. One of these methods is a type of therapy with a low-power diode laser, known as low-level laser therapy (LLLT) or photobiomodulation (PBM). It has been demonstrated that it can reduce chronic pain and may also have anti-inflammatory effects [8,9,10]. In this therapy, light is used to stimulate the cellular response, promote the healing of tissues, and reduce inflammation and pain [10]. It has been suggested that its anti-inflammatory effect comes from the inhibition of the cyclooxygenase cascade and the reduction in the synthesis of prostaglandins [9,10,11].

Despite its potential, the efficacy of LLLT is still controversial, as it is influenced by the placebo effect, and the variability in the laser parameters affects the energy intensity, application time, and the modality of application (intraoral or extraoral) [12]. Pre-clinical studies suggest that LLLT could improve the bone healing process and accelerate osseointegration, but the results from randomized clinical trials are inconsistent [13]. A recent systematic review indicated that LLLT was effective in reducing pain, edema, and trismus after wisdom tooth surgery, defining it as a viable option for the management of the postoperative inflammatory process as a simple and relatively fast non-pharmacological method [14]. However, additional systematic reviews reveal contradictory data and considerable heterogeneity in the studies. A lack of consensus about the optimum PBM protocol for the osseointegration of dental implants may be due to the different devices and application protocols utilized [13].

Studies such as the one by Memarian et al. [15] have highlighted that therapy with LLLT and LEDs can not only improve the stability of the patient, but can also help reduce inflammation markers, which may be associated with a decrease in postoperative pain. Clinical studies such as the one by Caccianiga et al. [16] demonstrated that the subjects in the experimental group showed a significant reduction in the facial swelling score as compared to the control and placebo groups, which corroborates the use of PBM to manage postoperative pain and suggests it has a role in facilitating vasodilation, improving circulation, phagocytosis, and lymphatic drainage.

Given the need to clarify these findings, more clinical research is imperative to assess methods that can be utilized in the treatment of postoperative pain and inflammation. Thus, the hypothesis of the present study is that PBM can efficiently modulate the inflammatory process generated during the surgical treatment and improve postoperative comfort. Thus, the objective of the present study is to quantitatively measure pain and inflammation values and to compare the proposed PBM treatment with a simulated treatment.

## 2. Method

### 2.1. Recruitment and Characteristics of the Patients

The study was designed as a simple, comparative, controlled, randomized clinical trial, in a single center that followed the requisites from the CONSORT declaration and the guidelines from the Helsinki Declaration. The study obtained a favorable report by the Bioethics Committee from the University of Murcia (ID: 2934/2020). It was conducted at a private dental clinic in the Region of Murcia (Spain). The study was registered at ClinicalTrials.gov, with the following identification number: NCT06535035.

Inclusion criteria: Older adult patients who needed rehabilitation with dental implants and who signed informed consent.

Exclusion criteria: Patients who, according to the ASA1y 2 preoperatory assessment, did not meet the medical conditions for a surgical intervention. Immunocompromised patients. Patients with decompensated systemic diseases; patients undergoing chemotherapy; or immunosuppressed treatments. Pregnant women. Patients with severe mental disorders. Patients who had received radiotherapy in their heads and necks.

### 2.2. Study Groups and Sample Size

A total of 62 patients were included and randomized into two groups with the use of the free online tool OxMar (Oxford Minimization and Randomization) [17], which was carried out by an external individual. Simple randomization was performed for both study groups. Group 1 (Experimental): A total of 31 patients were subjected to the insertion of dental implants with an immediate intraoral PBM session with an EPIC X Biolase diode laser (BIOLASE, Inc., Foothill Ranch, CA, USA), with a voltage of 100–240 V, 1.5 A, and a power of 0.5 W, and the application of 15 J per cm^2^, for 10–30 s at 1 mm from the tissue with a sterile surgical tip, based on the optimum biostimulation parameters by Cronshaw et al. [18]. The laser was applied to three different locations in the area, the buccal side, palatal/lingual side, and occlusal side, to stimulate tissue. Group II (Placebo): A total of 31 patients were subjected to implant surgery, after which, an inactive/simulated PBM with the same procedure was applied.

Sample size: Given the absence of previous data, the determination of the study sample size was performed with the following assumptions: an alpha risk of 0.05 (95% confidence) and a beta risk of 0.2 (power of 0.8) in a two-tailed contrast for the comparison of means between two independent groups, assuming unknown but equal variances and to determine an effect size of 0.80 (*d* = 0.8). Thus, the number of patients was estimated to be 25 subjects per group. Lastly, 20% more patients were used in each of the groups due to their possible loss. Thus, the sample size of the study was composed of a total of 60 patients (30 per group).

### 2.3. Procedure

#### 2.3.1. First Session

The patients were included in the study after meeting all the eligibility criteria and signing the informed consent form to participate and to allow the use of the data obtained for research aims. The following tests and questionnaires were used:

- Complete clinical and oral assessment.
- Plaque index evaluation by Löe and Silness [19]. Good hygiene was considered with a value of zero in this index, and hygiene was considered to be bad when the values were 1, 2, and 3.
- Modified Dental Anxiety Scale by Corah (MDAS) [20,21].
- Oral Health Impact Profile Questionnaire (OHIP-14sp) [22].
- Radiological study based on cone beam computed tomography (CBCT) before the surgery.

In the surgical intervention, performed by the first operator (F.P.P.), a specialist in oral surgery and implants with more than 10 years of experience, Straumann^®^, Basel, Switzerland, and Nobel^®^, Kloten, Switzerland, brand implants with an internal connection were placed. The number and location of the implants were assessed, as well as the duration of the surgery through the use of a digital chronometer. LLLT was applied by the second operator (Y.C.M.) according to the study group. Lastly, post-surgery instructions were given about oral hygiene, antibiotic guidelines (750 mg/8 h of Amoxicillin for 7 days), and analgesics (600 mg/8 h of ibuprofen for 4 days). Also, a questionnaire with a visual analog scale (VAS) was provided to assess inflammation and pain during the 7 days after the treatment.

#### 2.3.2. Second Visit (Monitoring and Suture Removal) after 7 Days

**Figure 1:**
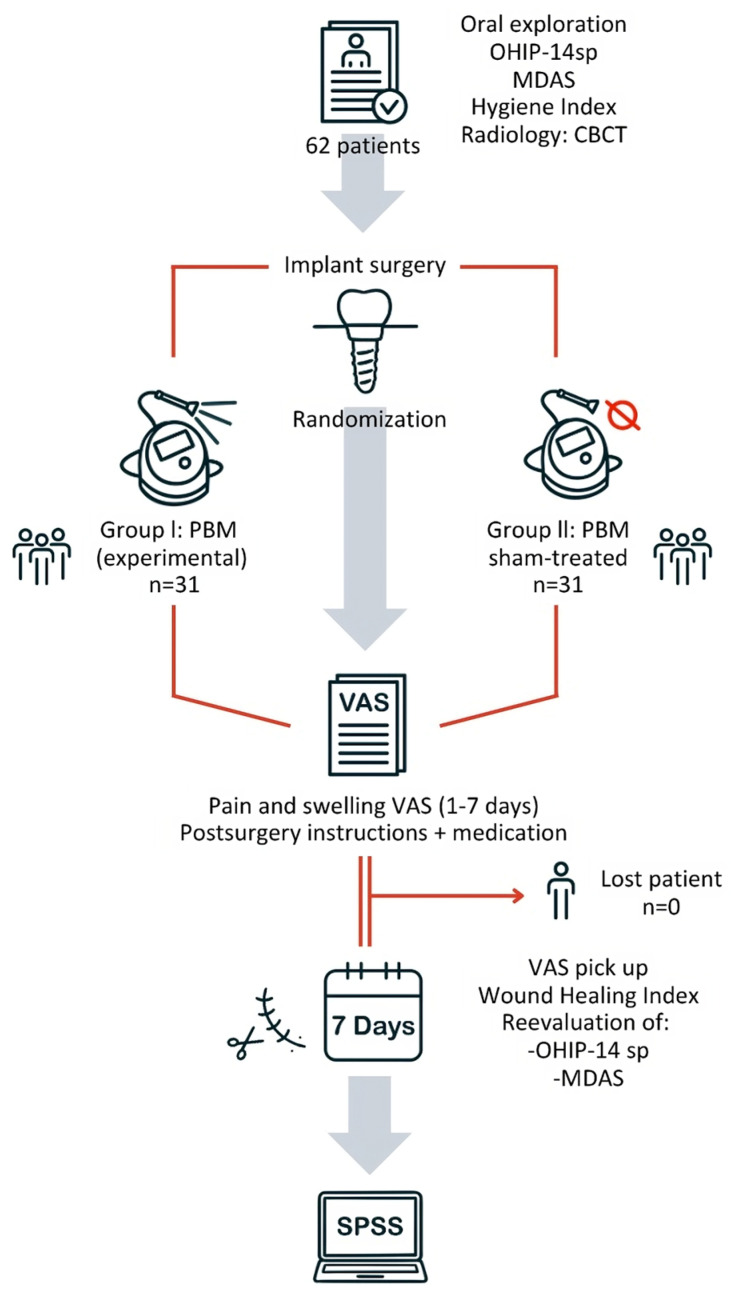
Scheme of the study design.

**Figure 2:**
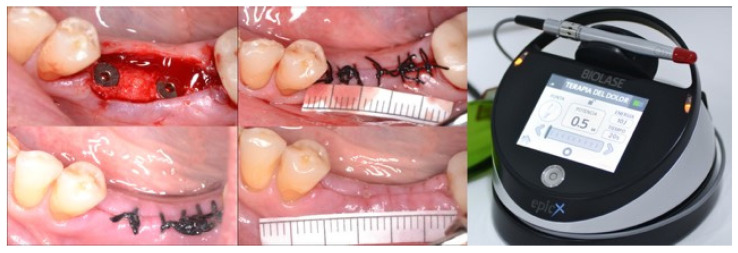
Top left: Intraoperative placement of two implants. Top right: Suture placement after surgery. Bottom left: Status of the suture after one week. Bottom right: Suture removal. BIOLASE EpicX, Foothill Ranch, CA, USA, diode laser device with the Pain Therapy program loaded.

- Collection of pain and inflammation questionnaires.
- Assessment of the healing rate.
- Re-evaluation of the MDAS scale and OHIP-14sp questionnaire (Figure 1).

- Assessment of postoperative pain: Postoperative pain was assessed 24 h, 48 h, and 7 days after the surgery by using a visual analog scale (VAS) with scores ranging from 0 to 10 (0 indicates “no pain”, and 10, the worst pain possible).
- Post-surgery inflammation was assessed by the patients themselves through a VAS 24h, 48h, and 7 days after the procedure, with scores ranging from 0 to 10 (0, a lack of inflammation, and 10, maximum inflammation).
- Plaque index by Löe and Silness. The plaque index by Löe and Silness was used on day 1 to assess the patient’s hygiene (0 = no plaque, 1 = no plaque visible, but observed when probing, 2 = plaque visible, and 3 = visible plaque, interproximal and/or presence of calculus) [19]. To simplify this index, a value of 0 was used as good hygiene, and the rest of the values were used to indicate bad hygiene, as bacterial plaque was observed.
- MDAS scale for dental anxiety. The Modified Dental Anxiety Scale (MDAS) by Corah was used to grade anxiety before the dental treatment [16,17]. (0, relaxed; 1, slightly anxious; 2, quite anxious; 3, very anxious; or 4, extremely anxious). A total score was obtained, and 4 ranges of fear and anxiety were established (<9, slight or no anxiety; 9–12, moderate anxiety; 13–14, high anxiety; >14, phobia) [20,21].
- Another variable was the OHIP-14sp questionnaire, which was used on day 0 and after 7 days. It consisted of 14 questions related to different dimensions of the quality of life of the patient (functional limitation, pain, psychological discomfort, physical disability, psychological disability, social disability, and handicap), with scores ranging from 0 to 5 for each question, and a maximum total score of 70. A higher score indicated the worse quality of life of the patient [22].
- Healing was calculated through the use of the healing index (HI) according to Hamzani and Chaushu (2018) [23], with a final score that varied between 1 and 5, 1 for poor healing and 5 for excellent healing, as a function of the behavior of the tissue (suppuration, pain, infection, slow healing, or suture dehiscence (Figure 2)).

### 2.4. Statistical Analysis

The quantitative variables were described through the use of means and standard deviations, while the qualitative ones were described through absolute and relative frequencies. The comparisons between groups were performed with Student’s *t* or Chi-square tests. Mixed effects models were created to determine the existence, or not, of pain and inflammation maintained over time, and if any of the factors (group, location of the implant site, hygiene index, number of implants, and MDAS) had an influence on them. The statistical analysis was performed with the SPSS 27.0 program for Windows. The differences were considered statistically significant if *p* < 0.05.

## 3. Results

The final study sample was composed of 62 patients, of which, 58.1% (*n* = 36) were men and 41.9% (*n* = 26) women, with ages ranging between 23 and 76 years, with a mean age of 53.4 years (SD = 14.3). No significant differences were observed between treatment groups in the demographic and clinical variables (Table 1).

**Figure 3:**
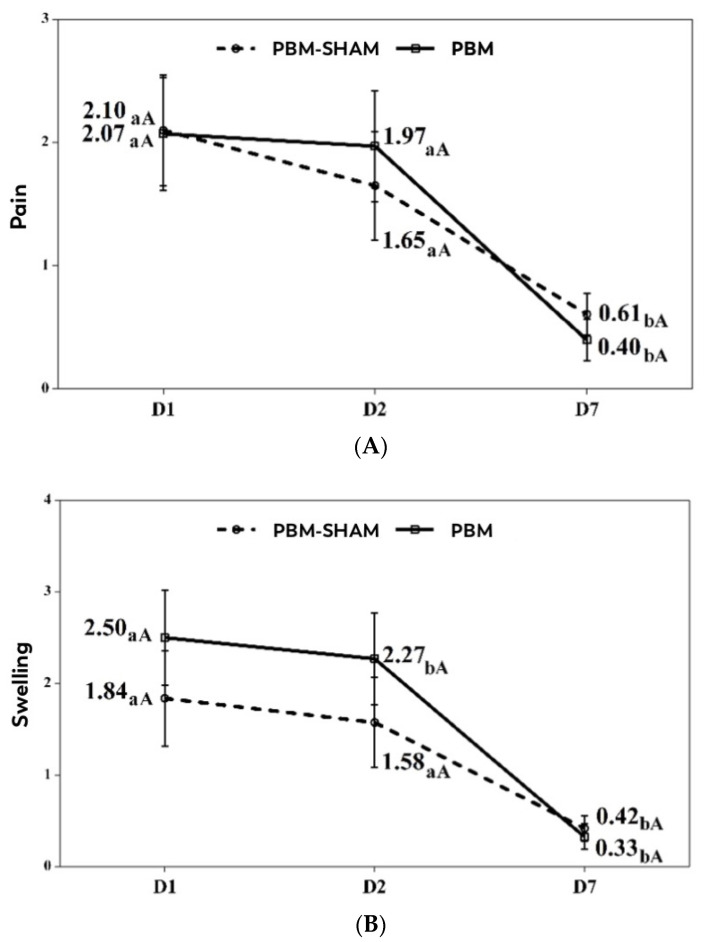
(**A**,**B**) Changes in pain and inflammation scores by group a–b. Pairwise comparisons between days. Different lowercase letters indicate statistically significant differences between days in the same group (Bonferroni correction). A–A. Pairwise comparisons between groups. Different uppercase letters indicate statistically significant differences between groups in the same assessment (Bonferroni correction).

Table 2 shows the results from the repeated-measures, two-factor ANOVA performed to determine the effect of the treatment on pain and inflammation. For both pain and inflammation, the results showed that the effect of time since the surgery was statistically significant, indicating that their values significantly decreased throughout the assessments, independently of the treatment. The interactions of time with the PBM treatment group or the simulated treatment were not statistically significant, which indicates that the changes produced in pain and inflammation were not influenced by the treatment. Figure 3A,B show the changes in the pain and inflammation values in each of the treatment groups.

**Figure 4:**
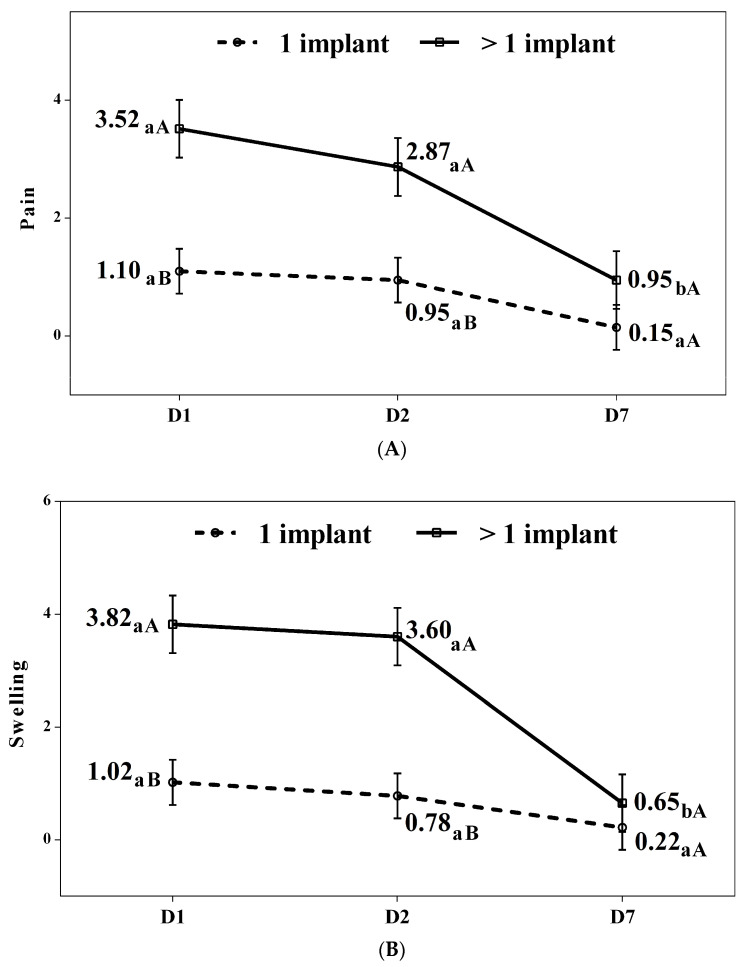
(**A**,**B**) Pain and inflammation scores according to the number of implants per group. A–b. Pairwise comparisons between days. Different lowercase letters indicate statistically significant differences between days in the same group (Bonferroni correction). A–B. Pairwise comparisons between groups. Different capital letters indicate statistically significant differences between groups at the same assessment (Bonferroni correction).

The results from the mixed effects models created are shown below. These were carried out to determine the existence of pain and inflammation over time, and if any of the factors (PBM group or simulated treatment/PBM-SHAM group, location of implant site, hygiene index, number of implants, and MDAS) had an influence on it. With respect to pain (Table 3), it was observed that the model was significant (*p* < 0.001), and that the significant effects were time and the interaction between time and the number of implants. The time effect indicates that pain significantly changed throughout the study, independently of the remaining variables. But the significant effect of the interaction between time and the number of implants indicates that the passing of time had a different influence on the patients, depending on the number of implants (Figure 4).

For the patients with a single implant, the pain remained without statistically significant changes throughout the study, while for patients with more than one implant, the pain significantly decreased after 7 days as compared to day 1 (*p* = 0.001) and day 2 (*p* = 0.019).

Regarding the comparison between patients based on the number of implants placed, those with a single implant experienced less pain on days 1 (*p* < 0.001) and 2 (*p* = 0.002). However, by day 7, no significant differences were observed compared to the group with more than one implant (*p* = 0.198)

With respect to inflammation (Table 4), it was observed that the model was significant (*p* < 0.001) and that the significant effects were the length of time that had passed since the surgery and the interaction between time and the number of implants. The time effect indicates that the inflammation changed significantly throughout the study, independently of the rest of the variables. However, the significant effect of the interaction of time and the number of implants indicates that time had a different influence on the patients, depending on the number of implants (Figure 4A,B).

The inflammation in patients with a single implant was lower than in the group with more than one implant on both day 1 (*p* < 0.001) and day 2 (*p* < 0.001). However, by day 7, no significant differences were observed between the groups (*p* = 0.504).

## 4. Discussion

Pain and inflammation are common postoperative complications after surgical procedures in the oral cavity, so their management is crucial for the success of the treatment [1,2,3,4,5]. In our study, through the use of mixed effects, we found that the time that had passed between the surgery and the number of implants were the most important variables that had an influence on the postoperative pain and inflammation. No adverse effects were observed in any of the study groups.

González-Santana et al. (2003) analyzed 41 patients and 131 implants, assessing pain and inflammation without the application of any photobiomodulation therapy. They found that pain reached its peak 6 h after the surgery, and they also found a significant relationship between pain and the number of implants. Inflammation reached its maximum after 48 h and was also significantly related with the number of implants, an advanced age, and the complexity of the interventions [2]. Our study also found a significant relationship between the maintenance of pain and inflammation and the number of implants placed, which suggests the need for effective strategies to manage these postoperative symptoms. To address this need, diverse studies have explored the application of photobiomodulation. Safdari et al. (2018) measured pain after implant surgery up to 72 h and found a significant reduction in pain and its intensity in the experimental group, which was treated with a laser, as compared to the placebo and control groups. Also, the patients in the experimental group showed a reduction in facial inflammation until day 3, which suggests that the intensity of pain and inflammation is stronger in the first few days [24].

In a posterior study, Pouremadi et al. (2019) [25] conducted a triple-blind study with 30 patients subjected to advance implant surgery. These authors compared a group treated with a laser and a placebo group, and found that the levels of pain and facial inflammation were significantly lower in the laser-treated group at days 3 and 7 post-surgery. Also, the healing of the wounds was better in the laser-treated group, so the study concluded that LLLT improved healing and reduced pain and inflammation after implant surgery [25]. Similarly, Caccianiga et al. (2020) used a single laser session after the surgery and recorded pain and inflammation through a VAS. They observed a significant decrease in both parameters in the experimental group as compared to the control and placebo groups, indicating a faster recovery in the laser-treated group [16].

In a recent publication from 2024, Basualdo Allende et al. (2024) [26] found that a single dose of 22.4 J of LLLT reduced postoperative pain from the implant surgery after 24 h in partially edentulous patients and after 24 and 72 h in patients who were completely edentulous. These findings are consistent with our results, indicating that the associated pain is influenced by factors such as the number of implants and the length of time since the surgery [26].

In the present study, we found that both pain and inflammation decreased in both groups, PBM and PBM-SHAM, but without statistically significant differences. The variables of length of time since the surgery and the number of implants had the most influence on these results. In the studies reviewed, great variability was observed in the PBM application protocols (intraoral or extraoral), the patient population, and the characteristics of the population. These differences can cause significant variations in the results, limiting the comparability of the findings. Also, the deficiencies in the research methods and the limited number of participants affected the reliability of the assays.

Another interesting aspect of PBM is its action on osseointegration. Camolesi et al. (2023) conducted a randomized double-blind study about photobiomodulation on the stability of dental implants and postoperative healing. LLLT was underlined due to its regenerative effects and the improvement in microvascular circulation in the treated site [27].

The meta-analysis performed by Sourvanos et al. (2023) supports the use of PBM after the placement of titanium implants. These authors observed significant improvements in the stability of the implants with diverse PBM devices. The amount of time spent in the application of PBM oscillated between 3 and 1200 s. A significant correlation was not found between the application time and the stability of the implant due to the heterogeneity of the results, wavelengths, clinical protocols, and implant dimensions [28].

Future studies must compare similar treatment protocols (wavelength, laser power, fluence), the placement of the implant and size, as well as the density of the bone tissue, intraoral application or extraoral, the alveolus ridge, and surrounding tissue to determine the optimum prescriptions, as these factors directly affect the depth of penetration and the amount of light received. The need for greater standardization in the clinical protocols in order to optimize the results is underlined.

As a limitation, we must underline that the assessment of pain and inflammation was performed subjectively by the patient. As previously mentioned, this can be greatly variable among individuals, depending on the individual’s sensitivity and the heterogeneity of the sample. Another significant limitation in our study was the lack of the assessment of the stability of the implant at different times using standardized methods. This lack of assessment limits our ability to make solid conclusions on the sustained efficacy of the LLLT or PBM in the maintenance of the long-term stability of implants.

## 5. Conclusions

Photobiomodulation (PBM) is a non-invasive and safe treatment. In our study, we found that postoperative pain and inflammation associated with implant surgery decreased similarly over time, independently of the application of PBM. These results suggest that PBM, although promising, did not show significant differences as compared to the simulated treatment (PBM-SHAM) in our study conditions. Therefore, more randomized studies are needed with a standardized methodology to adequately assess the efficacy of this therapy.

## Figures and Tables

**Table 1:** Demographic and clinical characteristics according to group.

	Group		*p*
	SHAM (*n* = 31)	PBM (*n* = 31)	
Sex			0.607
Man	17 (54.8)	19 (61.3)	
Woman	14 (45.2)	12 (38.7)	
Age	55.83 (13.37)	50.97 (14.91)	0.196
Length of surgery (minutes)	20.87 (8.89)	25.13 (10.58)	0.098
N. of implants			0.430
1	21 (67.7)	18 (58.1)	
More than 1	10 (32.3)	13 (41.9)	
Arch			0.611
Superior	16 (51.6)	14 (45.2)	
Inferior	15 (48.4)	17 (54.8)	
Hygiene INDEX			0.138
Good	10 (32.3)	5 (16.1)	
Bad	21 (67.7)	26 (83.9)	
OHIP TOTAL (DAY 1)	14.87 (26.21)	17.13 (27.24)	0.741
MDAS TOTAL (DAY 1)	5.94 (5.07)	5.32 (4.89)	0.629
HEALING INDEX			0.52
Poor healing	3 (9.7)	1 (3.2)	
Bad healing	3 (9.7)	4 (12.9)	
Good healing	2 (6.5)	3 (9.7)	
Very good healing	4 (12.9)	4 (12.9)	
Excellent healing	19 (61.3)	17 (54.8)	
Missing values	0	2 (6.5)	

**Table 2:** Descriptive values and statistical comparisons.

	Measurement			Intra-Subject Effects	
	D1	D2	D7	Time	Time * Group
	*Mean (SD)*	*Mean (SD)*	*Mean (SD)*	*F*(gl); *p*-Value (*η*^2^)	*F*(gl); *p*-Value (*η*^2^)
Pain				*F*(2;80) = 29.09; *p* < 0.001 (0.33)	*F*(2;80) = 0.76; *p* = 0.471 (0.013)
Sham	2.10 (2.31)	1.65 (2.11)	0.61 (1.02)		
PBM	2.07 (2.72)	1.97 (2.75)	0.40 (0.86)		
Inflammation				*F*(2;80) = 29.10; *p* < 0.001 (0.33)	*F*(2;80) = 1.48; *p* = 0.231 (0.025)
Sham	1.84 (2.42)	1.58 (2.22)	0.42 (0.81)		
PBM	2.50 (3.27)	2.27 (3.16)	0.33 (0.76)		

gl: degrees of freedom. *η*^2^: partial eta squared (effect size).

**Table 3:** Pain mixed effects model.

Origin	F	gl1	gl2	Sig.
Corrected model	4.37	7	175	<0.001
Time	12.09	1	175	0.001
Time * Group	0.26	1	175	0.613
Time * Site of placement	0.40	2	175	0.672
Time * Hygiene index	0.38	1	175	0.538
Time * N. of implants	8.54	1	175	0.004
Time * MDAS	0.06	1	175	0.807

gl: Degrees of freedom.

**Table 4:** Inflammation mixed effects model.

Origin	F	gl1	gl2	Sig.
Corrected model	5.95	6	176	<0.001
Time	15.64	1	176	<0.001
Time * Group	0.07	1	176	0.798
Time * Site of placement	0.07	2	176	0.929
Time * Hygiene index	0.34	1	176	0.559
Time * N. of implants	11.96	1	176	0.001

gl: degrees of freedoms.

## Data Availability

The data presented in this study are available on request from the corresponding author.
